# Antiviral Effects of *Lindera* *obtusiloba* Leaf Extract on Murine Norovirus-1 (MNV-1), a Human Norovirus Surrogate, and Potential Application to Model Foods

**DOI:** 10.3390/antibiotics9100697

**Published:** 2020-10-14

**Authors:** Diana Solis-Sanchez, Adriana Rivera-Piza, Soyoung Lee, Jia Kim, Bomi Kim, Joo Bong Choi, Ye Won Kim, Gwang Pyo Ko, Moon Jung Song, Sung-Joon Lee

**Affiliations:** 1 Department of Biotechnology, School of Life Sciences and Biotechnology for BK21 PLUS, Korea University, Seoul 02841, Korea; solis.sanchez.diana@gmail.com (D.S.-S.); adri.ripiz@gmail.com (A.R.-P.); sotting88@naver.com (S.L.); dreamjia@korea.ac.kr (J.K.); yppup@nate.com (B.K.); joobong90@gmail.com (J.B.C.); fink96@korea.ac.kr (Y.W.K.); 2 Institute of Health and Environment, Department of Environmental Health, Center for Human and Environmental Microbiome, Graduate School of Public Health, Seoul National University, Seoul 151-742, Korea; gko@snu.ac.kr; 3 Virus-Host Interactions Laboratory, Department of Biosystems and Biotechnology, Division of Biotechnology, College of Life Sciences and Biotechnology, Korea University, Seoul 02841, Korea; moonsong@korea.ac.kr

**Keywords:** anti-noroviral activity, *Lindera obtusiloba* leaf extract, MNV-1, β-pinene

## Abstract

Noroviruses are the leading cause of acute gastroenteritis and food poisoning worldwide. In this study, we investigated the anti-noroviral activity of *Lindera obtusiloba* leaf extract (LOLE) using murine norovirus (MNV-1), a surrogate of human norovirus. Preincubation of MNV-1 with LOLE at 4, 8, or 12 mg/mL for 1 h at 25 °C significantly reduced viral infectivity, by 51.8%, 64.1%, and 71.2%, respectively. Among LOLE single compounds, β-pinene (49.7%), α-phellandrene (26.2%), and (+)-limonene (17.0%) demonstrated significant inhibitory effects on viral infectivity after pretreatment with MNV-1, suggesting that the anti-noroviral effects of LOLE may be due to the synergetic activity of several compounds, with β-pinene as a key molecule. The inhibitory effect of LOLE was tested on the edible surfaces of lettuce, cabbage, and oysters, as well as on stainless steel. After one hour of incubation at 25°C, LOLE (12 mg/mL) pretreatment significantly reduced MNV-1 plaque formation on lettuce (76.4%), cabbage (60.0%), oyster (38.2%), and stainless-steel (62.8%). These results suggest that LOLE effectively inhibits norovirus on food and metal surfaces. In summary, LOLE, including β-pinene, may inactivate norovirus and could be used as a natural agent promoting food safety and hygiene.

## 1. Introduction

Noroviruses are the leading cause of acute gastroenteritis and food poisoning worldwide. It is estimated that norovirus infections are responsible for 200,000 deaths annually, including approximately 70,000 child deaths in developing counties [1,2]. Substantial progress has been made in the prevention of norovirus outbreaks. Nevertheless, gastroenteritis caused by norovirus infection remains the fourth leading cause of morbidity, and the second leading cause of illness, in children under five years of age worldwide [3]. While norovirus infections typically cause mild and self-limiting symptoms lasting 24–48 h, persistent, chronic infections can cause severe symptoms [4]. Although potential norovirus vaccines have been developed, no specific treatment is currently available due to the high mutation rate during viral replication [5,6].

Noroviruses were first identified in elementary school students with symptoms of gastroenteritis in Norwalk (OH, USA) using immune electron microscopy [7]. Since then, at least 33 norovirus genotypes have been identified. Noroviruses that infect humans belong to the GI, GII, and GIV genogroups. Variants of the GII.4 genotype are the most common cause of norovirus illness worldwide, accounting for 70–80% of the highly infective global norovirus outbreaks [8]. Recently, outbreaks of previously rare genotypes, such as GII.17 and GII.2, have occurred, and were responsible for most of the norovirus cases in some part of the world [8]. Due to the limitations of the propagation methods for human norovirus, in in vitro and animal models, viral surrogates such as murine norovirus (MNV-1) are widely used to predict human norovirus behavior, and have also been used in food safety research [9,10]. MNV-1 is remarkably stable and can endure harsh conditions such as low pH, freezing temperatures, thermal inactivation, and exposure to organic solvent/alcohol-based disinfectants; moreover, it is persistent on surfaces under dry and wet conditions [4,10]. MNV-1 is also easy to culture, and thus has been employed in several norovirus inactivation studies [11,12,13,14].

Norovirus is highly contagious, sheds rapidly and prolifically in feces, and has a high mutation rate. Hence, inactivation of norovirus is critical to prevent further outbreaks [15,16]. Several inactivation strategies have been used; however, physical methods such as heat exposure and UV radiation require specific instrumentation, while chemical treatments such as surface disinfectants, sanitizers, and ozone treatment cannot completely inactivate norovirus [17,18]. Furthermore, prolonged and continuous use of synthetic substances and disinfectants can be highly hazardous to human health and the environment.

Plant-derived natural substances have potential antimicrobial and antiviral effects against a wide range of food pathogens and can be used in food safety applications [19]. Moreover, several studies have demonstrated the anti-noroviral activity of phytochemicals and its bioactive compounds, with low or no toxicity [12,20].

*Lindera obtusiloba* is a natural edible herb used in East Asian countries, including Korea, Japan, and China [21]. The extracts of this plant have been used in Korean traditional medicine for the treatment of liver and inflammatory diseases, and poor blood circulation [22]. *Lindera obtusiloba* also show antibacterial activity against foodborne pathogens, including *E. coli* O157: H7, *Salmonella typhimurium, Staphylococcus aureus,* and others [21]. In this study, we investigated the potential antiviral activity of *Lindera obtusiloba* leaf extract (LOLE) using an MNV-1 surrogate system. We also explored the effect of key single compounds in LOLE on MNV-1 inhibition, and the inhibitory effects of LOLE on MNV-1 in model foods and on steel surfaces.

## 2. Results

### 2.1. Evaluation of LOLE Cytotoxicity

**Figure 1:**
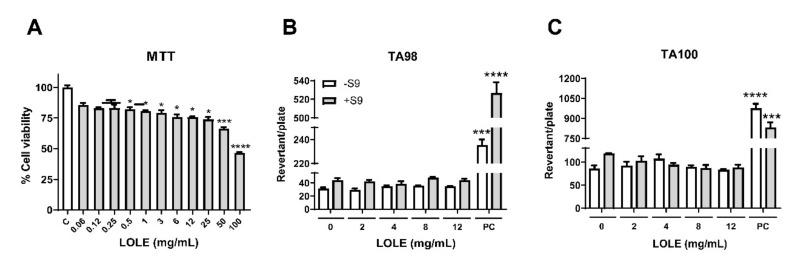
Safety assessment of *Lindera obtusiloba* leaf extract (LOLE) by cytotoxicity and mutagenicity assays. (**A**) The percentage of cell viability was determined by MTT assay. RAW 264.7 cells were treated with LOLE at various concentrations, and formazan absorbance was measured at 570 nm. The relative cell viability was compared to the control treated with DMSO. (**B**,**C**) Evaluation of LOLE by Ames test with strains TA98 and TA100, without (S9+) and with metabolic activation (S9+). The positive controls (PC) employed for (S9+) were 4-NQO (4-nitroquinoline 1-oxide, 15 μg per plate) and for (S9+), 2-AA (2-aminoanthracene, 5 μg per plate), respectively. Phosphate-buffered saline (PBS)-treated samples were used as negative controls for the test without metabolic activation. The white bars represent the control groups, whereas the gray bars represent the intervention groups. Results are expressed as the percentage of neutralized virus compared to control (mean ± SEM; n = 3). **** *p* < 0.0001 *** *p* < 0.001, * *p* < 0.05 vs. negative control.

A safety assessment of *Lindera obtusiloba* leaf extract (LOLE) was performed to investigate whether LOLE exerts potential cell cytotoxicity by MTT assay in RAW 264.7 murine macrophages (Figure 1A). LOLE showed no significant cytotoxic effects up to 0.25 mg/mL. The genotoxicity of LOLE was also evaluated using an Ames mutagenic assay and TA98 and TA100 strains with (S9+) and without (S9+) metabolic activation (Figure 1B,C). The results showed no mutagenic effect of LOLE in S9+ and S9+ compared to negative controls. In vitro toxicity assay results suggested that LOLE is an edible herb extract with potential anti-noroviral activity and no significant cytotoxicity in RAW 264.7 cells.

### 2.2. Dose-Response and Incubation Times of LOLE on Neutralization of MNV-1

**Figure 2:**
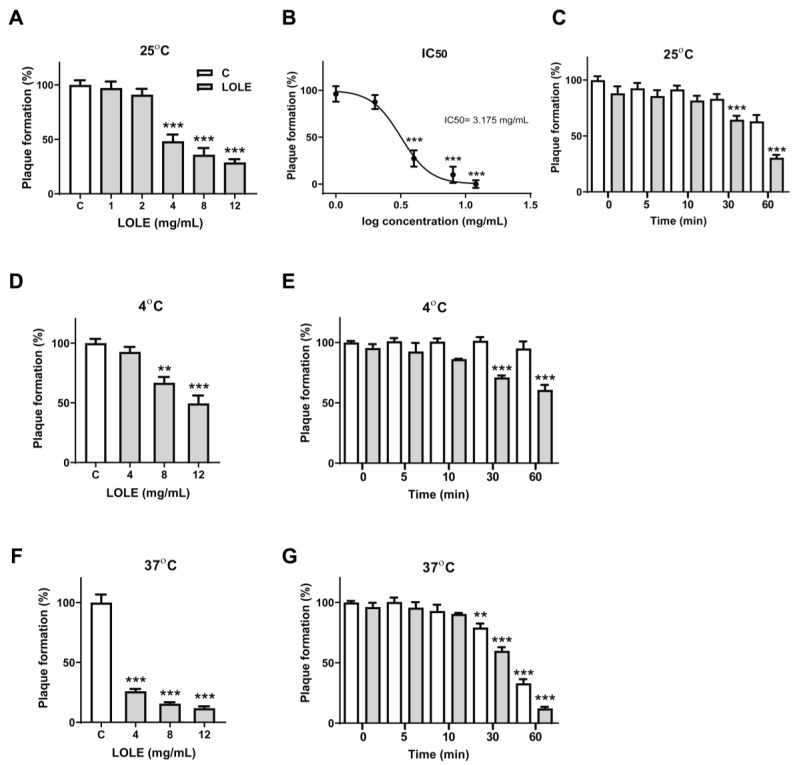
Temperature-, dose-, and time-response relationships between *Lindera obtusiloba* leaf extract (LOLE) exposure and murine norovirus (MNV-1) inactivation in RAW 264.7 cells. MNV-1 virus particles were pretreated with LOLE solutions of 1, 2, 4, 8, and 12 mg/mL for 1 h at 25 °C before infecting RAW 264.7 cells. Dimethyl sulfoxide (DMSO) was used as a control and virus titer was measured by plaque assay. (**A**,**B**) Effect of LOLE on plaque formation (%) at various concentrations. (**C**) MNV-1 virus particles were pretreated for 0, 5, 10, 30 and 60 min with LOLE (12 mg/mL) at 25 °C. (**D**,**F**) For temperature-dependent experiments, MNV-1 virus particles were pretreated with LOLE solutions of 4, 8, and 12 mg/mL for 1 h at 4 °C and 37 °C. For the time- and temperature-dependent experiments, virus particles were pretreated for 0, 5, 10, 30 and 60 min with LOLE (12 mg/mL) at 4 °C and 37 °C (**E**,**G**). The mean diameter of the plaques was 1 mm, and visible plaques were counted 72 h after infection (triplicate determinations). The white bars represent the control groups, whereas the gray bars represent the intervention groups. Results are expressed as the percentage of neutralized virus compared to control (mean ± SEM; n = 3). ** *p* < 0.01, *** *p* < 0.001 compared to control.

To assess the anti-noroviral effect of LOLE, the human norovirus surrogate MNV-1 was preincubated with increasing concentrations of LOLE (1–12 mg/mL) for 1 h at 25°C. The neutralized virus was quantified by plaque assay. The anti-noroviral activity of LOLE is described in Figure 2A. The results showed that the MNV-1-neutralizing effect of LOLE was dose-dependent at concentrations above 4 mg/mL, with plaque formation reductions of 51.8% (4 mg/mL), 64.1% (8 mg/mL) and 71.2% (12 mg/mL). LOLE significantly reduced MNV-1 infectivity with an IC_50_ of 3.175 mg/mL (Figure 2B). To determine the impact of incubation period on anti-noroviral effectiveness, MNV-1 was preincubated with 12 mg/mL of LOLE at 25°C for 0, 5, 10, 30, and 60 min (Figure 2C). The results showed that MNV-1 infectivity was reduced significantly after 30 min (35.5%) and 60 min (69.5%). The results suggest that LOLE may exert profound anti-noroviral effects in a dose- and time-dependent manner.

### 2.3. Temperature-, Dose-, and Time-Response Relationships between LOLE Exposure and MNV-1 Inactivation

It has been reported previously that temperature plays a key role in the survival of MNV-1 in food over time [23]. Therefore, we performed additional plaque reduction assays to investigate the effects of LOLE on MNV-1 inactivation at 4 °C and 37 °C. The results showed that, at 4 °C, pretreatment of MNV-1 with LOLE significantly reduced plaque formation, by 33.2% (8 mg/mL) and 50.5% (12 mg/mL) (Figure 2D). At 37 °C (Figure 2F), the reduction of plaque formation was greater, at 74.1% (4 mg/mL), 84.4% (8 mg/mL), and 88.1% (12 mg/mL).

Time-dependent incubation experiments were performed concurrently at 4 °C and 37 °C to examine the anti-noroviral activity effect of LOLE (12 mg/mL). LOLE significantly reduced plaque formation at 4 °C after 30 min (29.0%), and 60 min (39.2%) (Figure 2E). Similar results were observed at 37 °C, when plaque formation was also reduced significantly after 30 min (40.1%), and 60 min (87.7%) (Figure 2G). Interestingly, LOLE failed to reduce plaque formation with shorter pretreatment periods (<30 min). Collectively, the results suggested that pretreatment of MNV-1 with LOLE neutralizes MNV-1 in a dose- and time-dependent manner at 37 °C.

### 2.4. Anti-Noroviral Activity of LOLE Single Compounds

**Figure 3:**
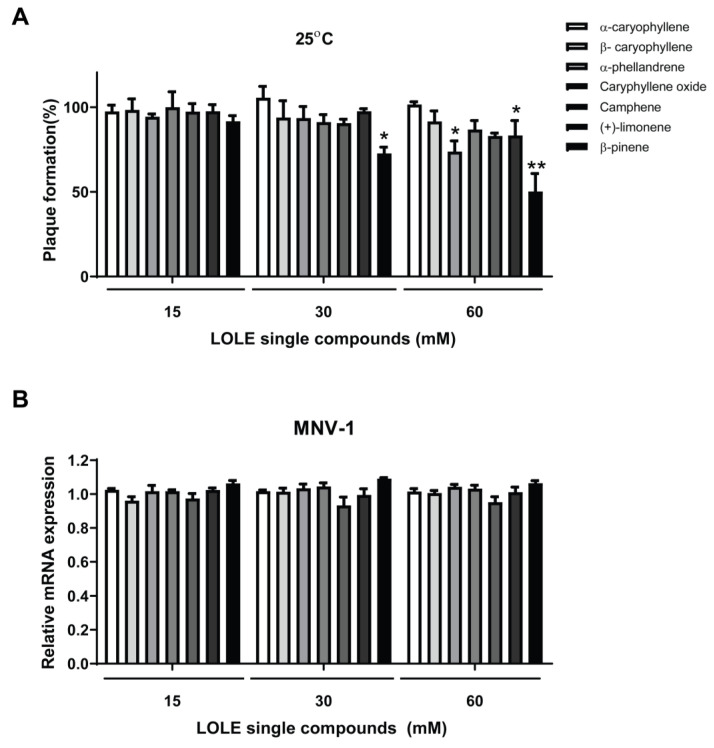
Anti-noroviral activity of *Lindera obtusiloba* leaf extract (LOLE) single compounds against murine norovirus (MNV-1). MNV-1 virus particles were preincubated with 15, 30 and, 60 mM LOLE solutions for 1 h at 25 °C before infection of RAW 264.7 cells. (**A**) The effect on plaque formation (%) of LOLE compounds was evaluated by plaque reduction assays. MNV-1 infectivity was calculated as a percentage of plaque formation using a dimethyl sulfoxide (DMSO)-treated control as a reference. (**B**) Quantification of viral mRNA of MNV-1 in infected RAW 264.7 cells. Gene expression is shown as fold-change relative to the expression in control (glyceraldehyde 3-phosphate dehydrogenase; GAPDH). The white bars represent the control groups, whereas the gray bars represent the intervention groups. Results are expressed as the percentage of neutralized virus compared to control (mean ± SEM; n = 3). * *p* < 0.05, ** *p* < 0.01 vs. DMSO.

To further explore the anti-noroviral activity of LOLE, seven key compounds therein were selected to assess its effect on MNV-1 inhibition. The anti-noroviral activities of α-caryophyllene, β-caryophyllene, α-phellandrene, caryophyllene oxide, camphene, (+)-limonene and β-pinene were evaluated by plaque reduction assays (Figure 3A). Among the tested compounds, α-phellandrene (26.2%), (+)-limonene (17.0%), and β-pinene (49.7%,) at 60 mM significantly reduced plaque formation after 1 h of incubation at 25°C. Among the tested LOLE single compounds, α-phellandrene, (+)-limonene, and β-pinene showed the greatest MNV-1 inhibitory effect. Conceivably, there may be synergetic effects among these three compounds.

### 2.5. LOLE Single Compounds Did not Significantly Reduce MNV-1 Genome Copies

The anti-noroviral activity of LOLE may be related to three major biological processes: attachment, internalization, and replication. Substances that interfere with the successful completion of any of these phases may help inhibit viral infection. Hence, to determine if key LOLE single compounds reduced MNV-1 infectivity by inhibiting virus adsorption in RAW 264.7 cells, the number of MNV-1 genome copies was quantified by qPCR. MNV-1 was preincubated with the single compounds contained in LOLE at concentrations of 15, 30, and 60 mM (Figure 3B). DMSO-treated virus samples were used as controls. The results suggested that pretreatment of the MNV-1 with key single LOLE compounds did not prevent MNV-1 adsorption in RAW 264.7 cells.

### 2.6. Inactivation of Norovirus by LOLE in Model Food Systems

**Figure 4:**
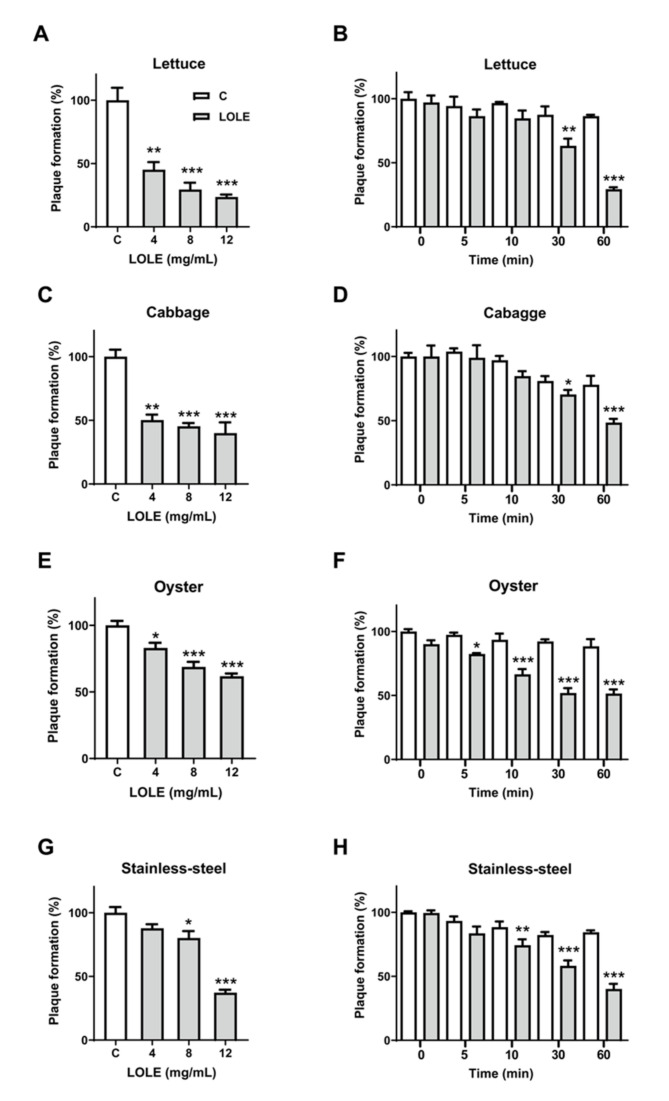
*Lindera obtusiloba* leaf extract (LOLE) anti-noroviral effects on model food systems. Lettuce, cabbage, oyster and stainless-steel surfaces were spot-inoculated with murine norovirus (MNV-1) (10^5^ PFU/mL) and plaque reduction assays were performed to determine the anti-noroviral effects of LOLE. (**A**,**C**,**E**,**G**) Dose-response experiments: MNV-1 virus particles were pretreated with LOLE solutions (1, 2, 4, 8, and 12 mg/mL) for 1 h at 25 °C before infecting RAW 264.7 cells. (**B**,**D**,**F**,**H**) Time-response experiments: virus particles were pretreated with LOLE (12 mg/mL) for 0, 5, 10, 30 and 60 min at 25 °C. MNV-1 infectivity was calculated as a percentage of plaque formation using dimethyl sulfoxide (DMSO)-treated samples as a control. The white bars represent the control groups, whereas the gray bars represent the intervention groups. Results are expressed as the percentage of neutralized virus compared to control (mean ± SEM; n = 3). * *p* < 0.05, ** *p* < 0.01, *** *p* < 0.001 vs. DMSO.

The inhibitory effect of LOLE on norovirus was assessed on several known carriers identified in previous norovirus outbreaks: lettuce, cabbage, oysters, and a stainless-steel surface (Figure 4). A significant dose-dependent reduction of plaque formation was observed in all samples pretreated with LOLE (Figure 4A, 4C, 4E, and 4G). After 1-hour incubation at 25 °C, pretreatment with 12 mg/mL LOLE significantly reduced MNV-1 plaque formation in lettuce (76.4%), cabbage (60.0%), oyster (38.2%), and stainless-steel (62.8%). Thus, the anti-noroviral effect of LOLE was greatest on lettuce surfaces. LOLE (12 mg/mL) showed a significant MNV-1-inhibiting effect after 30-min incubation in all model systems (Figure 4B, 4D, 4F, and 4H). These results suggest that MNV-1 could be effectively inhibited on lettuce, cabbage, oyster, and stainless-steel surfaces by exposure to LOLE for 30 min.

## 3. Discussion

Natural compounds derived from plants have been widely used in traditional medicine due to their therapeutic properties. Several studies have attributed antibacterial and antiviral properties to these substances, which can be used to preserve food quality and extend food product shelf-life [24]. As a response to increasing consumer awareness regarding food safety, natural compounds have emerged as an attractive alternative to ensure the quality of food products [25]. Recent studies have also examined the effects of natural substances and plant-based essential oils against norovirus infectivity [4,12,20,23]. For instance, previous studies reported that extracts from *Lindera obtusiloba* have antibacterial effects against various foodborne pathogens, including *E. coli* O157: H7, *Salmonella typhimurium,* and *Staphylococcus aureus,* among others [21]. We investigated the neutralizing effect of LOLE and its key single compounds on MNV-1 under various temperature, dose and time conditions.

A safety assessment showed that LOLE had no significant cytotoxic effects up to 0.25 mg/mL, suggesting that it could be used to assess anti-noroviral activity without significant cytotoxicity in RAW 264.7 cells. Hong et al. (2009) reported no adverse effects of LOLE consumption in Sprague-Dawley rats fed with the extract (2000 mg/kg daily) for 14 days [26]. Other studies focusing on the therapeutic properties of LOLE using in vivo models did not report any negative effects of the extract on the health of the animals [27]. Gilling et al. [28] reported significant reductions of MNV with application of essential oils from oregano, clove and Zataria. The reductions in that study appeared to be temperature-dependent.

Preincubation of the norovirus with natural substances can be used to determine the capacity of these compounds to reduce infectivity. Incubation of MNV with LOLE was found to significantly inhibit subsequent infections in a mouse macrophage cell line, in a time- and dose-dependent manner. Temperature appeared to be another critical factor. LOLE exhibited a greater effect on MNV-1 neutralization at 37 °C compared to 4 °C. These observations are consistent with the results reported by Elizaquível et al. [29], which suggested that temperature is an important factor in the stability and infectivity of the norovirus, and that lower temperatures may compromise MNV-1 stability. Also, time-dependent experiments showed that the reduction observed in cell culture infectivity for MNV-1 increased with longer LOLE exposure periods. Therefore, LOLE anti-noroviral activity at 4 °C may be improved by longer pretreatment incubation periods, potentially leading to similar anti-noroviral results to those obtained at 37 °C.

LOLE is a complex substance that contains a wide variety of active compounds. In this study, seven representative compounds contained in the plant extract were assessed by plaque reduction assay, to identify the substance responsible for the anti-noroviral activity of LOLE. The compounds were as follows: α-caryophyllene, β-caryophyllene, α-phellandrene, caryophyllene oxide, camphene, (+)-limonene and β-pinene [30,31]. Previous studies reported significant antimicrobial effects of these compounds against a wide range of pathogens. Some of these pathogens cause foodborne diseases, which suggests that they may also possess antiviral activity [12,14,23,32]. The mechanisms by which antimicrobials exert their antiviral effect remains unclear. Some studies have suggested that antimicrobials may act upon the virus capsid or block receptors, which in the case of nonenveloped viruses, such as MNV-1, can protect the integrity of the viral genetic information and facilitate virus adsorption to receptors in the host cell [20,25,33].

Among the selected compounds, α-phellandrene, (+)-limonene, and β-pinene showed significant plaque formation reduction ability at 60 mM. MNV-1 RNA relative gene expression was assessed by qPCR to determine if the reduction in plaque formation was due to the inhibition of viral adsorption to the host. Pretreatment of MNV-1 with LOLE single compounds did not significantly reduce the number of MNV-1 gene copies, suggesting that no significant damage occurred. Although the virus particles appeared to be intact after pretreatment with the selected compounds, it is possible that the particles had become inactivated (non-infectious). Inactivation may be another mechanism by which single compounds exert inhibitory activity against MNV-1, besides viral particle degradation. For example, compounds may bind to the virus capsid and block the epitopes required for specific adsorption of MNV-1 to the host cells. Alternatively, they may cause a conformational change of the virus capsid, resulting in nonspecific adsorption [34]. Further experiments are required to determine the mechanism underlying the anti-noroviral activity of LOLE compounds.

Non-thermal processed food and fresh produce have been increasingly associated with foodborne norovirus outbreaks [25]. Unprocessed food can be contaminated by contact with food pathogens in irrigation water, and during handling or improper washing. Several enteric viruses that are responsible for foodborne diseases can survive in harsh environments, and even a few particles can cause illness [35]. In the food industry, the treatment of fresh fruit and vegetables is often inadequate to eliminate pathogens. Typical treatments include submersion in chlorine. Although this procedure has previously been shown to be effective for eliminating enteric pathogens, it has limited to no effect on viral pathogens [15,18,36]. In a previous investigation, Di Caprio et al. [37]. tested the enteric virus disinfection efficiency of 200 ppm chlorine treatment on romaine lettuce roots and shoots, and green onion roots. The results indicated limited or no reduction in the viral titer of MNV-1 and Tulane virus, which is another human norovirus surrogate.

It has been reported that some components of the food matrix can limit the efficiency of physical and chemical treatments for norovirus inactivation and inhibition [17,38]. Therefore, it is important that the anti-noroviral activity of new treatments is not affected by the food matrix. Fresh oyster, lettuce, and cabbage have been identified as norovirus carriers during foodborne outbreaks, along with food preparation surfaces (e.g., stainless-steel surfaces) [39]. In this study, we evaluate the antiviral effect of LOLE against cultivable human norovirus surrogates in model foods including lettuce, cabbage, oyster, and on stainless-steel surfaces. The results showed that MNV-1 pretreatment with LOLE significantly attenuated subsequent infections in a mouse macrophage cell line, in a time- and dose-dependent manner. 

## 4. Materials and Methods

### 4.1. Material and Reagents

LOLE was obtained from the Korean Plant Extract Bank (No. 026-052, 2004/10/20). Based on previous studies, seven key LOLE compounds (α-caryophyllene, β-caryophyllene, α-phellandrene, camphene, caryophyllene oxide, (+)-limonene and β-pinene) were analyzed to assess its effect on MNV-1 inhibition [31,40]. β-caryophyllene, α-phellandrene, camphene, caryophyllene oxide, and β-pinene were purchased from Sigma-Aldrich (St. Louis, MO, USA); α-caryophyllene was from Santa Cruz Biotechnology (Santa Cruz, CA, USA) and (+)-limonene was from Acros Organics (Morris Plains, NJ, USA). LOLE and single compounds were dissolved in dimethyl sulfoxide (DMSO; Bio Basic Inc., Markham, ON, Canada) and stored at −20 °C. When used in further experiments, compounds were dissolved in serum-free Dulbecco’s modified Eagle’s medium (DMEM; Hyclone, Logan, UT, USA) to appropriate concentrations. MNV-1 was kindly provided by Dr. Herbert Virgin (Washington University, St. Louis, MO, USA).

RAW 264.7 cells were obtained from the Korean Cell Line Bank (Seoul, Republic of Korea) and cultured in DMEM that included 10% fetal bovine serum (FBS, Hyclone, Logan, UT, USA), 10 mM non-essential amino acids, 10 mM HEPES (*N*-2-hydroxyethylpiperazine-*N*’-2-ethane sulfonic acid), 10 mM sodium bicarbonate and 1% gentamicin (Gibco, Grand Island, NY, USA). RAW 264.7 cells were maintained at 37 °C in a 5% CO_2_ humidified incubator and sub-cultured every 2–3 days to avoid cell activation.

### 4.2. 3-[4,5-Dimethylthiazol-2-yl]-2,5-Diphenyltetrazolium Bromide (MTT) Cell Viability Assay

The viability of LOLE was measured using the colorimetric MTT assay (Sigma-Aldrich, St. Louis, MO, USA) as previously described [23]. Briefly, RAW 264.7 cells were seeded into 96-well culture plates (SPL Life Sciences, Gyeonggi-do, Korea) at a density of 8 × 10^4^ cells/mL and incubated overnight at 37 °C in an atmosphere of 5% CO_2._ LOLE was diluted in series in serum-free DMEM media, as follows: 100, 50, 25, 12, 6, 3, 1, 0.5, 0.25, 0.125, and 0.0625 mg/mL. RAW 264.7 cells were treated with 200 µL of each concentration and incubated for 10 min. After media removal, 200 µL of culture medium containing 10% MTT was added to each well and incubated for 3 h at 37 °C. The MTT solution was then removed and the cells were dried for 1 min by inverting the plate. Then, 200 μL of DMSO was added to each well to dissolve the formazan crystals. The solution was carefully resuspended and the absorbance was measured at 570 nm using a microplate reader (Multiskan GO, Thermo Fisher Scientific, Waltham, MA, USA).

### 4.3. Mutagenic Ames Test

Mutagenic activity was tested using a *Salmonella* assay and *Salmonella typhimurium* tester strains TA98 and TA100 (Xenometrix AG, Allschwil, Switzerland)., with and without metabolization according to Maron and Ames’ preincubation method [41]. In brief, TA98 reverse mutations are induced by a frameshift mutagen, while TA100 reverse mutations are induced by a mutagen that first induces a base-pair substitution at one GC-site [41]. The mixture was freshly prepared before each test. For this analysis, 100 µL of an overnight culture of TA98 or TA100 (1–2 × 10^9^ cells/mL) was added to a 15-mL conical tube (SPL Life Sciences, Gyeonggi-do, Korea) and then treated with 4, 8 or 12 mg of LOLE dissolved in 1× phosphate-buffered saline (PBS). For assays without metabolic activation (S9+), the following mixture was used: 5 mM D-glucose-6-phosphate, 4 mM nicotinamide adenine dinucleotide phosphate, 16 mM magnesium chloride, 100 mM sodium phosphate dibasic, and 100 mM sodium phosphate monobasic (Sigma-Aldrich, St. Louis, MO, USA). For metabolic activation assessment (S9+), 66 mM potassium chloride (Bio Basic Inc., Markham, ON, Canada) was used. The tubes were incubated at 37 °C in a shaking incubator (200 rpm) for 45 min. Following incubation, 2 mL of top agar (0.6%) (Bacto Aga, BD, Franklin Lakes, NJ, USA) containing 0.5% sodium chloride (Duksan Reagents, Seoul, South Korea) and 0.05 mM L-histidine and 0.05 mM biotin (Sigma-Aldrich, St. Louis, MO, USA) was added to each tube at 45 °C, promptly mixed and poured into prepared Petri dishes containing 1.5% minimal agar medium (Junsei, Tokyo, Japan) and 0.5% glucose (Sigma-Aldrich, St. Louis, MO, USA) in Vogel-Bonner E medium. The Petri dishes were incubated for 48 h at 37 °C, and revertant colonies (His+) were counted. PBS was used as a negative control, 4-nitroquinoline 1-oxide (4-NQO; 15 μg per plate) (Sigma-Aldrich, St. Louis, MO, USA) and 2-aminoanthracene (2-AA; 5 μg per plate) (Sigma-Aldrich, St. Louis, MO, USA) were used as positive controls for the test without (S9-) and with metabolic activation (S9+), respectively.

### 4.4. Preparation and Titration of MNV-1

For MNV-1 stock preparation, a monolayer of RAW 264.7 cells in a 175-cm2 cell culture dish (SPL Life Sciences, Gyeonggi-do, Korea) was infected with MNV-1 at a multiplicity of infection (MOI) of 0.01 in a volume of 5 mL serum-free DMEM [42]. Cells were incubated at 37 °C in a 5% CO2 atmosphere with agitation every 15 min for 1 h. The viral inoculum was removed, and cells were washed with serum-free DMEM. Then, complete medium was added to the cell culture dish, and cells were incubated at 37 °C in 5% CO2 for 48 h or until the viral-induced cytopathic effect was observed. The virus-infected cells were frozen at −80 °C overnight and thawed on ice to obtain cell lysate before viral purification. The cell lysate was collected, mixed with an equal volume of chloroform, and filtered using an Amicon Ultra-15 filter with a molecular weight cutoff of 10 kDa (Millipore, Billerica, MA, USA) at 5000 rcf for 20 min. The concentrated MNV-1 suspension was aliquoted and stored at -80 °C until further use.

To determine the viral concentration, 2 × 106 cells/well RAW 264.7 were seeded into a 6-well culture plate (SPL Life Sciences, Gyeonggi-do, Korea) and incubated overnight at 37 °C in a 5% CO2 atmosphere. The concentrated MNV-1 suspension was thawed and serially diluted from 1:103 to 1:108 in serum-free DMEM. Then, the cells were infected with 500 μL of the MNV-1 dilutions, and 500 μL of DMEM serum-free medium was added to each well. The plates were incubated at 37 °C for 1 h, with agitation every 15 min. The viral suspension was removed, and cells were overlaid with 3 mL of 1.5% SeaPlaque agarose (Lonza, Rockland, ME, USA) mixed with an equal volume of 2 × MEM complete media (Sigma-Aldrich, St. Louis, MO, USA) and incubated at 37 °C for 48 h. The cells were fixed, stained, and incubated (6 h at 37 °C) in an atmosphere of 5% CO2 to count the number of plaques. The stain consisted of 3 mL of a 1% neutral red solution (Sigma-Aldrich, St. Louis, MO, USA) dissolved in 1.5% SeaPlaque agarose mixed with an equal volume of 2 × MEM complete media. Plaques per well were counted and used to calculate the virus titer and determine the PFU/mL.

### 4.5. Plaque Reduction Assay

RAW 264.7 cells (2 × 10^6^ cells/well) were seeded into a 6-well culture plate and incubated overnight at 37 °C in an atmosphere of 5% CO_2_. A suspension of 10^5^ PFU/mL MNV-1 particles in serum-free DMEM was pretreated with LOLE or single compounds previously diluted in serum-free DMEM at various concentrations (1, 2, 4, 8, and 12 mg/mL for LOLE; 15, 30 mM and 60 mM for single compounds). Viral suspensions were diluted 1:100 with serum-free DMEM, and 500 μL of the suspension was added to each well. The plates were incubated at 37 °C for 1 h, with agitation every 15 min. After MNV-1 infection, the medium was removed, and cells were overlaid with 3 mL of 1.5% SeaPlaque agarose (Lonza, Basel, Switzerland) and mixed with an equal volume of 2 × MEM complete media (Sigma-Aldrich, St. Louis, MO, USA) [43]. After 48 h of infection, the cells were fixed and stained with 3 mL of 1% neutral red solution (Sigma-Aldrich) dissolved in 1.5% SeaPlaque agarose mixed with an equal volume of 2 × MEM complete media. After an incubation of 6 h, plaque formation was analyzed. DMSO at the previously mentioned dilutions was used as the control. Antiviral activity was expressed as the percentage (%) of plaque formation compared to the control group. The 50% inhibitory concentration (IC_50_) was calculated using GraphPad Prism 5 software (GraphPad Software, Inc., La Jolla, CA, USA), following the manufacturer’s instructions, i.e., using the inhibition results when MNV-1 was pretreated with LOLE from 1–12 mg/mL at 25 °C.

### 4.6. Plaque Reduction Assay of MNV-1 on Model Foods and Stainless-Steel

The anti-noroviral effect of LOLE was examined on model foods and stainless-steel. Experiments were carried out on sterile surfaces of lettuce and cabbage (2 × 2 cm) and oyster (1 × 1 cm). Briefly, sections were cut and washed in sterile triple-distilled water, submerged into 70% ethanol for 1 min, and rinsed with sterile triple-distilled water. Similarly, 2 × 2 cm stainless-steel sections were sterilized as previously mentioned. All samples were air-dried in sterile Petri dishes in a biosafety cabinet. MNV-1 (10^5^ PFU/mL) was spot-inoculated on the sample surfaces and air-dried for 5–10 min to allow virus attachment. To determine the effects of LOLE on MNV-1 infectivity, the surface was spot-treated with 4, 8, or 12 mg/mL of LOLE diluted in serum-free DMEM and incubated at 25 °C for 1 h. A concentration of 12 mg/mL of LOLE was selected to assess the effect of incubation period on MNV-1 infectivity; the effects were measured at 0, 5, 10, 30, and 60 min. For virus recovery, the samples were transferred into a 50-mL conical tube containing 0.5 g of glass beads (0.5 mm) (DAIHAN Scientific, Wonju, Korea) and 1 mL of serum-free DMEM. Each tube was vigorously vortexed for 30 s to improve virus elution from the surface via mechanical abrasion. A plaque reduction assay was performed as described in the methods.

### 4.7. Assessment of MNV-1 Viral Adsorption on Host Cells by Quantitative Polymerase Chain Reaction (qPCR)

MNV-1 at 0.1 MOI [44] was preincubated with the compounds at different concentrations (15, 30, or 60 mM, diluted in DMEM serum-free media) for 1 h at 4 °C to determine if LOLE single compounds reduced norovirus infectivity by inhibiting virus adsorption to the host cell. Then, treatment suspensions were diluted up to 1 mL with DMEM serum-free media, and the nearly confluent RAW264.7 cells were infected with the treatment suspensions and incubated for 1 h at 37 °C. Subsequently, the inoculum was removed, and the cells were washed with 1 mL of PBS. The MNV-1-infected RAW264.7 cells were lysed by three cycles of freeze-thawing, and the cell lysates were collected to isolate total RNA using RNAiso PLUS (Takara, Shiga, Japan). cDNAs were synthesized from 0.5 μg of total RNA using oligo dT primer and ReverTra Ace qPCR RT Master Mix with gDNA Remover (Toyobo, Tokyo, Japan). The qPCR was performed using Thunderbird SYBR qPCR Mix (Toyobo, Tokyo, Japan) containing 2 μL of cDNA, 10 μL of SYBR master mix, 600 nM of each primer (forward and reverse), and 0.4 μL of ROX dye and diethyl pyrocarbonate (DEPC)-treated water up to a 20 μL reaction volume. PCR amplification was performed using an IQ5 real-time PCR detection system (Bio-Rad, Hercules, CA, USA) under the following conditions: initial denaturation at 95 °C for 3 min, followed by 50 cycles of amplification with denaturation at 95 °C for 20 s, annealing and elongation at 55 °C for 20 s, and 72 °C for 1 min. The data were analyzed using IQ5 Optical System Software (version 2; Bio-Rad, Hercules, CA, USA). The PCR primers used for MNV-1 analysis were 5′-ACGCCACTCCGCACAAA-3′ and 5′-GCGGCCAGAGACCACAAA-3′. The glyceraldehyde 3-phosphate dehydrogenase (GAPDH) gene was used as a reference to normalize the MNV-1 gene expression.

### 4.8. Statistical Analysis

All experiments were performed in triplicate. Student’s t-test and one-way ANOVA followed by Tukey’s test were used to compare two and more than two groups, respectively. Data are shown as mean ± standard error of the mean (SEM). *p <* 0.05 was considered as statistically significant.

## 5. Conclusions

In conclusion, our results demonstrated that LOLE may has potential to be used as an anti-noroviral agent, either as a food additive or surface sanitizer for the control and prevention of norovirus outbreaks. However, further studies are needed to identify the precise mechanism in which LOLE exerts its anti-noroviral activity.
